# Health-Related quality of life in Russian children with inflammatory bowel disease is comparable across disease types and treatment modalities

**DOI:** 10.3389/fped.2026.1908855

**Published:** 2026-07-29

**Authors:** Yuen Kai Lim, Alexandre V. Gorelov, Ekaterina A. Yablokova, Elena V. Borisova, Victoria S. Krikun, Svetlana B. Krutikhina, Irina V. Ozerskaia, Maria A. Kudryashova, Angelina V. Polyanskaya, Svetlana N. Chebysheva, Olga A. Savvateeva, Alexey M. Savvateev

**Affiliations:** 1 Department of Children's Diseases, Filatov Clinical Institute of Children's Health, Sechenov First Moscow State Medical University (Sechenov University), Moscow, Russia; 2 Central Research Institute of Epidemiology, Federal Service for Surveillance on Consumer Rights Protection and Human Wellbeing, Moscow, Russia; 3 Research Clinical Institute of Childhood, Ministry of Health of Moscow Region, Moscow, Russia

**Keywords:** biologic therapies, crohn's disease, inflammatory bowel diseases, health-related quality of life, ulcerative colitis

## Abstract

**Background:**

Pediatric inflammatory bowel disease (IBD), including Crohn's disease (CD) and ulcerative colitis (UC), may affect health-related quality of life (HRQoL) beyond clinical disease activity alone. Although biologic therapy has improved disease control, its relationship with disease-specific HRQoL, generic HRQoL, and family functioning in real-world cohort of children with IBD remains insufficiently defined. This study aimed to assess HRQoL and family functioning in children with IBD according to treatment modality and disease type.

**Methods:**

This single-center, cross-sectional study enrolled children aged 8–17 years with confirmed IBD at Sechenov University, between 2024 and 2026. Participants were stratified by treatment modality into those receiving biologic therapy in combination with conventional therapy and those receiving conventional therapy alone, and by disease type into CD and UC groups. Disease-specific HRQoL was assessed using IMPACT-III, generic HRQoL using the Pediatric Quality of Life Inventory 4.0 Generic Core Scales (PedsQL), and family functioning using the PedsQL Family Impact Module (PedsQL FIM). Nonparametric methods were used, and correlations were assessed using Spearman's correlation coefficient.

**Results:**

The cohort comprised 105 children with IBD, including 59 with CD and 46 with UC. Thirty-eight children received biologic therapy in combination with conventional therapy, and 67 received conventional therapy alone. No statistically significant differences were detected between treatment groups in total IMPACT-III, PedsQL, or PedsQL FIM scores. Similarly, HRQoL and family functioning did not differ significantly between children with CD and UC. Across treatment groups, social functioning had the highest scores, whereas emotional functioning was consistently among the lowest-scoring domains. In the PedsQL FIM, family relationships had the highest scores and worry had the lowest scores. Significant positive correlations were identified between IMPACT-III and PedsQL total scores (*ρ* = 0.599, *p* < 0.001), between IMPACT-III and PedsQL FIM total scores (*ρ* = 0.291, *p* = 0.003), and between PedsQL and PedsQL FIM total scores (*ρ* = 0.333, *p* < 0.001).

**Conclusion:**

In this Russian cohort of children with IBD, HRQoL and family functioning were similar across treatment groups and disease types. Emotional functioning and parental worry remained key challenges, highlighting the importance of routine HRQoL assessment and family-centered support.

## Introduction

Inflammatory bowel diseases (IBD), including ulcerative colitis (UC) and Crohn's disease (CD), are chronic relapsing disorders of the gastrointestinal tract characterized by immune-mediated inflammation of the intestinal wall (1). Despite similarities in clinical presentation, these conditions differ substantially in their pathogenesis, localization, and patterns of inflammation (2).

In recent years, increasing attention has been paid to the assessment of health-related quality of life (HRQoL) in children with IBD. The chronic course of the disease, the need for long-term therapy, and limitations in daily activities have a significant impact on physical, emotional, and social functioning in children (3). Several studies have shown that HRQoL in children with IBD may be impaired even in the absence of marked clinical disease activity, highlighting the important role of psychosocial factors (4). At the same time, the two major forms of IBD differ in disease course and prognosis. Crohn's disease is generally characterized by a more aggressive and complicated course, including the development of strictures and fistulas, and a higher likelihood of requiring surgical interventions. In contrast, ulcerative colitis is confined to the colon and less frequently necessitates surgery; however, children with Crohn's disease have a substantially higher lifetime risk of operative treatment (5, 6). These differences may influence children's perceptions of the disease and, consequently, their quality of life. However, previous studies have not demonstrated consistent differences in HRQoL between these disease types (4, 7). Instead, disease activity rather than disease type has been identified as the main determinant of HRQoL in children IBD, with additional contributions from psychosocial factors, including emotional well-being and family functioning (8, 9).

Quality of life in children with IBD has been widely studied across different regions, including Russia, several European countries (Croatia, Switzerland, Sweden, France, and Greece) (4, 7, 10–13), and Asian populations such as Malaysia (14). Overall, these studies highlight the important role of both clinical and psychosocial factors in shaping HRQoL outcomes in children with IBD. Conventional therapy for IBD includes 5-aminosalicylic acid (5-ASA) agents (mesalazine), immunomodulators (azathioprine), and glucocorticoids, aimed at inducing and maintaining remission (15, 16). However, these approaches do not always achieve adequate disease control and may be associated with adverse effects (17).

The introduction of biologic therapies, including anti–tumor necrosis factor (anti-TNF) agents (infliximab, adalimumab) and anti-integrin agents (vedolizumab), has significantly expanded treatment options and improved the ability to achieve sustained remission (18, 19). Currently, biologic therapy represents a key component of treatment in children with moderate-to-severe IBD [5]. Despite their proven clinical efficacy, the impact of biologic therapies on quality of life—particularly in children —remains an area of active investigation. While improved disease control may enhance HRQoL, the need for long-term treatment and regular medical follow-up may also impose additional psychological burden on children and their families (20).

Despite the growing body of evidence, the combined assessment of disease-specific HRQoL, generic HRQoL, and family functioning in relation to both disease phenotype and treatment modality remains insufficiently explored, particularly in real-world pediatric clinical settings. Therefore, this study aimed to comprehensively assess disease-specific and generic HRQoL in children with ulcerative colitis and Crohn's disease, to evaluate family functioning, and to examine the impact of treatment modality on HRQoL outcomes.

## Materials and methods

### Study design and participants

This was a single-center, observational, cross-sectional study conducted between 2024 and 2026 at the Department of Gastroenterology, Sechenov Center for Mother and Child Health, Sechenov University (Moscow, Russian Federation). The study included children aged 8–17 years with a confirmed diagnosis of IBD, including UC and CD.

Diagnoses were established in accordance with current Russian Federation clinical guidelines, with consideration of recommendations from the European Society for Paediatric Gastroenterology, Hepatology and Nutrition (ESPGHAN), based on clinical, laboratory, endoscopic, and histological findings (5, 6, 15, 16). All assessments were performed during inpatient care.

Children were stratified according to disease type (UC or CD) and treatment modality at the time of HRQoL assessment. Treatment allocation was not randomized and was not determined by the study investigators, but reflected real-world clinical decision-making. Group 1 included children receiving biologic therapy in combination with conventional therapy for at least 3 months before inclusion, while Group 2 included children receiving conventional therapy alone for at least 3 months before inclusion and were not receiving biologic therapy at the time of questionnaire completion.

Treatment was administered in accordance with Russian Federation clinical guidelines, taking into account ESPGHAN recommendations. Conventional therapy included 5-ASA agents, immunomodulators, and glucocorticoids, while biologic therapy included anti-TNF agents, such as infliximab and adalimumab, and the anti-integrin agent vedolizumab, prescribed according to clinical indications. Escalation to biological therapy was considered in children with moderate to severe disease activity, high-risk disease phenotype, steroid dependency or resistance, inadequate response to conventional therapy or complicated disease course. Baseline comparability between groups was assessed based on demographic characteristics and disease activity indices [Pediatric Crohn's Disease Activity Index (PCDAI) and Pediatric Ulcerative Colitis Activity Index (PUCAI)] during the same hospitalization and reflected disease activity at the time of HRQoL questionnaire completion.

The study protocol was approved by the local independent ethics committee of Sechenov University (protocol No. 27-24, dated November 7, 2024). Written informed consent was obtained from all participants and/or their legal guardians prior to enrollment. Following consent, children independently completed the Pediatric Quality of Life Inventory 4.0 Generic Core Scales (PedsQL) and the IMPACT-III questionnaire in paper format, while their parents completed the PedsQL Family Impact Module (PedsQL FIM) on the same day. Data collection was performed at a single time point.

### Inclusion criteria

Participants were eligible for inclusion if they were aged 8–17 years and had a confirmed diagnosis of inflammatory bowel disease (ulcerative colitis or Crohn's disease). All children had received therapy for at least 3 months at the time of inclusion, either biologic therapy in combination with conventional therapy or conventional therapy alone. Eligible participants were required to be capable of independently completing patient-reported outcome questionnaires, including IMPACT-III and PedsQL, while their parent or legal guardian completed the PedsQL Family Impact Module (PedsQL FIM). Written informed consent was obtained from all participants and/or their legal guardians prior to enrollment.

### Exclusion criteria

Participants were excluded if they had severe somatic, infectious, or psychiatric conditions unrelated to inflammatory bowel disease that could influence the assessment of HRQoL. Additional exclusion criteria included significant cognitive impairment limiting the ability to understand and complete questionnaires, age below 8 years or above 17 years, therapy duration of less than 3 months, and absence of written informed consent. Participants were also excluded if relevant comorbid chronic conditions were identified during the study or if consent was withdrawn by the participant or their legal guardian.

### Sample size estimation

No *a priori* sample size calculation was performed. The sample comprised consecutively enrolled eligible children during the 2024–2026 study period. A *post hoc* sensitivity analysis was conducted for the main treatment-group comparison to estimate the minimum detectable standardized between-group difference at a two-sided *α* level of 0.05 and 80% power.

### Questionnaires

HRQoL was assessed using validated Russian versions of the IMPACT-III, PedsQL, and PedsQL FIM.

Disease-specific HRQoL was evaluated using the IMPACT-III questionnaire developed by Otley et al. (21, 22), comprising 35 items across four domains: well-being, emotional functioning, social functioning, and body image (23). Permission for its use was obtained via the Maritime Inflammatory Bowel Disease Research Alliance (MIRA, Canada). Generic HRQoL was assessed using the PedsQL 4.0 Generic Core Scales (Mapi Research Trust, Lyon, France) (24), which include 23 items covering physical, emotional, social, and school functioning. Family functioning was assessed using the PedsQL Family Impact Module (Mapi Research Trust) (25), consisting of 36 items across eight domains: physical, emotional, social, cognitive functioning, communication, worry, daily activities, and family relationships. All instruments have been previously validated for use in pediatric populations. Responses were recorded on a 5-point Likert scale and linearly transformed to a 0–100 scale (0 = 100, 1 = 75, 2 = 50, 3 = 25, 4 = 0). Higher scores indicated better HRQoL or more favorable family functioning. Domain and total scores were calculated as mean values.

### Statistical methods

Statistical analyses were performed using jamovi (version 2.5.7, The jamovi Project) and Microsoft Excel (2021). Normality was assessed using the Shapiro–Wilk test. Continuous variables were analyzed using nonparametric methods and are presented as median and interquartile range, with mean, standard deviation, and range reported additionally. Categorical variables are presented as counts and percentages and were compared using the *χ*^2^ test. Between-group differences in continuous variables were assessed using the Mann–Whitney *U*-test. Because multiple domain-level comparisons were performed across the HRQoL and family functioning instruments, false discovery rate (FDR) correction using the Benjamini–Hochberg procedure was applied separately to the treatment-modality comparisons and disease-type comparisons. Raw *p*-values are presented in the tables, and FDR-adjusted results were used to assess the robustness of domain-level findings.

An additional sensitivity analysis restricted to children with low clinical disease activity was performed for the total IMPACT-III, PedsQL, and PedsQL FIM scores. Because PCDAI and PUCAI are diagnosis-specific and are not directly interchangeable, low clinical disease activity was defined as PCDAI ≤10 for CD and PUCAI ≤10 for UC. Treatment groups were compared using the Mann–Whitney *U*-test. An exploratory diagnosis-stratified analysis compared treatment groups separately among children with CD and UC for total IMPACT-III, PedsQL, and PedsQL FIM scores using the Mann–Whitney *U*-test.

Correlations between HRQoL measures, family functioning, and disease activity indices were evaluated using Spearman's correlation coefficient (*ρ*). A two-sided *p*-value < 0.05 was considered statistically significant. Analyses were conducted using available data without imputation of missing values. An exploratory *post hoc* analysis examined correlations among the emotional-functioning domains of IMPACT-III, PedsQL, and PedsQL FIM. The resulting correlation matrix was visualized using a heatmap.

## Results

### Participant characteristics

A total of 105 children with IBD were included in the study, of whom 59 (56.2%) had CD and 46 (43.8%) had UC. The sex distribution was balanced, with a slight predominance of females (*n* = 54, 51.5%) compared to males (*n* = 51, 48.5%). The median age of children was 13.0 [10.0; 16.0] years. Children receiving biologic therapy were significantly older than those receiving conventional therapy (*p* = 0.024), while no significant differences in sex distribution were observed between the groups (*p* = 0.310). The distribution of disease types differed between treatment groups: Crohn's disease predominated in the biologic therapy group, whereas ulcerative colitis was more frequent in the conventional therapy group (*p* < 0.001).

Disease activity in children with Crohn's disease, assessed using PCDAI, was comparable between groups (*p* = 0.450). In contrast, among children with ulcerative colitis, disease activity measured by PUCAI was higher in the conventional therapy group (*p* = 0.011) (Table 1).

**Table 1:** Clinical and demographic characteristics of the single-center Russian cohort of children with IBD, stratified by treatment modality.

Variable	Group 1 (biologic therapy) (*n* = 38)	Group 2 (conventional therapy) (*n* = 67)	*p*-value
Age, years [Me (Q1; Q3)]	14.0 [12.3; 16.0]	12.0 [10.0; 16.0]	0.024**
Sex, *n* (%)			0.310*
Male	21 (55.3%)	30 (44.8%)	
Female	17 (44.7%)	37 (55.2%)	
Diagnosis, *n* (%)			<0.001*
Crohn's disease	29 (76.3%)	30 (44.8%)	
Ulcerative colitis	9 (23.7%)	37 (55.2%)	
PCDAI, Me [Q1; Q3]	10.0 [5.0; 15.0]	10.0 [10.0; 15.0]	0.450**
PUCAI, Me [Q1; Q3]	10.0 [5.0; 10.0]	15.0 [10.0; 30.0]	0.011**

* *χ*^2^ (chi-square) test for categorical variables.

** Mann–Whitney *U*-test for continuous variables.

PCDAI, Pediatric Crohn's Disease Activity Index; PUCAI, Pediatric Ulcerative Colitis Activity Index; IBD, inflammatory bowel disease.

### Post Hoc sensitivity analysis of sample size

With 38 children in the biologic-therapy group and 67 in the conventional-therapy group, the available sample size permitted detection of a standardized between-group difference of approximately Cohen's d = 0.57 at a two-sided *α* level of 0.05% and 80% power. This corresponds to a moderate minimum detectable effect; smaller between-group differences may not have been detected.

### Comparison of HRQoL: biologic vs. conventional therapy

#### IMPACT-III

No statistically significant differences in disease-specific HRQoL, as measured by the IMPACT-III questionnaire, were observed between children receiving biologic therapy and those receiving conventional therapy (Table 2). Children in the biologic therapy group demonstrated slightly higher median total scores [76.0 [64.8; 84.0] vs. 71.0 [61.0; 80.0]; *p* = 0.136] and higher median values in the well-being, emotional, and social functioning domains. In contrast, body image scores were comparable between groups. The largest between-group difference was observed in the emotional functioning domain [73.0 [58.0; 87.3] vs. 68.0 [48.0; 75.0]; *p* = 0.068], indicating a trend toward better emotional functioning in the biologic therapy group, although this did not reach statistical significance.

**Table 2:** Comparison of disease-specific HRQoL measured using IMPACT-III between children receiving biologic plus conventional therapy and those receiving conventional therapy alone.

IMPACT-III domains	Group 1 (biologic therapy) (*n* = 38) Me [Q1; Q3]	Group 2 (conventional therapy) (*n* = 67) Me [Q1; Q3]	*p*-value
Well-being	73.0 [63.0; 85.0]	69.0 [56.0; 79.0]	0.492
Emotional functioning	73.0 [58.0; 87.3]	68.0 [48.0; 75.0]	0.068
Social functioning	84.0 [75.0; 91.0]	80.0 [70.0; 89.0]	0.250
Body image	75.0 [64.5; 94.0]	81.0 [69.0; 88.0]	0.835
Total score	76.0 [64.8; 84.0]	71.0 [61.0; 80.0]	0.136

IMPACT-III, disease-specific HRQoL questionnaire for children with IBD; IBD, inflammatory bowel disease.

#### PedsQL

No statistically significant differences in generic HRQoL, as measured by the PedsQL questionnaire, were observed between Children receiving biologic therapy and those receiving conventional therapy (Table 3). Median total scores were identical in both groups (75.0), indicating comparable overall HRQoL. Scores across all domains were similar, including physical, emotional, social, and school functioning. Across both groups, the highest scores were observed in the social functioning domain (median 90.0), whereas the lowest scores were consistently found in the emotional functioning domain.

**Table 3:** Comparison of generic HRQoL measured using PedsQL 4.0 between children receiving biologic plus conventional therapy and those receiving conventional therapy alone.

PedsQL domains	Group 1 (biologic therapy) (*n* = 38) Me [Q1; Q3]	Group 2 (conventional therapy) (*n* = 67) Me [Q1; Q3]	*p*-value
Physical functioning	75.0 [66.0; 88.0]	78.0 [66.0; 88.0]	0.916
Emotional functioning	67.5 [50.0; 83.8]	60.0 [50.0; 80.0]	0.320
Social functioning	90.0 [75.0; 100.0]	90.0 [80.0; 100.0]	0.999
School functioning	65.0 [46.3; 80.0]	65.0 [50.0; 80.0]	0.213
Total score	75.0 [63.3; 87.5]	75.0 [64.5; 83.0]	0.962

PedsQL, Pediatric Quality of Life Inventory 4.0 Generic Core Scales.

#### PedsQL FIM

No statistically significant differences in family functioning, as assessed by PedsQL FIM, were observed between children receiving biologic therapy and those receiving conventional therapy (Table 4). Median total scores were comparable between groups [74.0 [54.3; 86.8] vs. 72.0 [60.0; 80.5]; *p* = 0.756]. Scores across all domains were similar. The highest scores were observed in the family relationships domain (median 90.0 in both groups), whereas the lowest scores were found in the worry domain (median 55.0 in both groups).

**Table 4:** Comparison of family functioning measured using PedsQL FIM between children receiving biologic plus conventional therapy and those receiving conventional therapy alone.

PedsQL FIM domains	Group 1 (biologic therapy) (*n* = 38) Me [Q1; Q3]	Group 2 (conventional therapy) (*n* = 67) Me [Q1; Q3]	*p*-value
Physical functioning	69.0 [54.0; 91.0]	67.0 [52.0; 79.0]	0.554
Emotional functioning	65.0 [50.0; 80.0]	55.0 [50.0; 75.0]	0.527
Social functioning	88.0 [64.5; 100.0]	81.0 [69.0; 94.0]	0.586
Cognitive functioning	80.0 [56.3; 95.0]	75.0 [65.0; 95.0]	0.881
Communication	67.0 [50.0; 92.0]	75.0 [67.0; 96.0]	0.206
Worry	55.0 [36.3; 73.8]	55.0 [45.0; 70.0]	0.717
Daily activities	83.0 [58.0; 100.0]	75.0 [50.0; 92.0]	0.239
Family relationships	90.0 [75.0; 100.0]	90.0 [75.0; 100.0]	0.671
Total score	74.0 [54.3; 86.8]	72.0 [60.0; 80.5]	0.756

PedsQL FIM, PedsQL Family Impact Module.

To account for multiple domain-level comparisons across IMPACT-III, PedsQL, and PedsQL FIM, FDR correction was applied to the treatment-modality comparisons. After correction, no domain-level differences between the biologic therapy and conventional therapy groups remained statistically significant.

### Sensitivity analysis restricted to children with low clinical disease activity

Because PUCAI values were significantly higher in children with ulcerative colitis receiving conventional therapy, a sensitivity analysis was performed in children with low clinical disease activity. Low clinical disease activity was defined as PCDAI ≤10 for Crohn's disease and PUCAI ≤10 for ulcerative colitis. This subgroup included 57 children, of whom 28 received biologic therapy and 29 received conventional therapy. No statistically significant differences were observed between treatment groups in IMPACT-III total score, PedsQL total score, or PedsQL FIM total score (Table 5).

**Table 5:** Sensitivity analysis comparing total HRQoL and family-functioning scores between treatment groups among children with low clinical disease activity.

Variable	Group 1 (biologic therapy) (*n* = 28) Me [Q1; Q3]	Group 2 (conventional therapy) (*n* = 29) Me [Q1; Q3]	Mann–Whitney U	*p*-value
IMPACT-III total score	76.0 [69.3; 84.0]	71.0 [60.0; 79.0]	302	0.098
PedsQL total score	75.0 [64.5; 82.8]	75.0 [61.0; 77.0]	352	0.393
PedsQL FIM total score	73.5 [58.8; 86.0]	75.0 [60.0; 83.0]	396	0.873

IMPACT-III, disease-specific HRQoL questionnaire for children with IBD; PedsQL, Pediatric Quality of Life Inventory 4.0 Generic Core Scales; PedsQL FIM, PedsQL Family Impact Module. Low clinical disease activity was defined as PCDAI ≤10 for CD and PUCAI ≤10 for UC.

Because disease type distribution differed significantly between treatment groups, an additional stratified analysis was performed by diagnosis. Treatment groups were compared separately among children with Crohn's disease and among children with ulcerative colitis for total IMPACT-III, PedsQL, and PedsQL FIM scores. In both disease strata, no statistically significant differences were observed between biologic therapy and conventional therapy groups in the total scores of the three HRQoL instruments (Table 6). However, these findings should be interpreted cautiously because of the limited subgroup size, particularly among children with ulcerative colitis receiving biologic therapy.

**Table 6:** Diagnosis-stratified comparison of total HRQoL and family-functioning scores between treatment groups among children with CD and UC.

Disease type	Variable	Group 1 (biologic therapy) Me [Q1; Q3]	Group 2 (conventional therapy) Me [Q1; Q3]	Mann–Whitney U	*p*-value
Crohn's disease	IMPACT-III total score	76.0 [64.0; 84.0]	72.5 [63.8; 79.0]	351	0.203
Crohn's disease	PedsQL total score	76.0 [65.0; 88.0]	71.5 [62.3; 82.3]	386	0.457
Crohn's disease	PedsQL FIM total score	74.0 [58.0; 87.0]	75.5 [59.3; 83.0]	430	0.940
Ulcerative colitis	IMPACT-III total score	76.0 [70.0; 80.0]	69.0 [61.0; 81.0]	141	0.489
Ulcerative colitis	PedsQL total score	66.0 [60.0; 80.0]	76.0 [66.0; 85.0]	140	0.471
Ulcerative colitis	PedsQL FIM total score	73.0 [53.0; 85.0]	71.0 [61.0; 78.0]	163	0.923

Between-group comparisons were performed using the Mann–Whitney *U*-test. The analysis was exploratory because of the limited subgroup size, particularly among children with ulcerative colitis receiving biologic therapy. IMPACT-III, disease-specific HRQoL questionnaire for children with IBD; PedsQL, Pediatric Quality of Life Inventory 4.0 Generic Core Scales; PedsQL FIM, PedsQL Family Impact Module.

### Comparison of HRQoL: Crohn's disease vs. ulcerative colitis

No statistically significant differences in HRQoL were observed between children with Crohn's disease and those with ulcerative colitis across all instruments (IMPACT-III, PedsQL, and PedsQL Family Impact Module) (Table 7). Median total scores were comparable between the two groups for all questionnaires, including IMPACT-III (76.0 vs. 70.5; *p* = 0.270), PedsQL (73.0 vs. 75.0; *p* = 0.681), and PedsQL FIM (75.0 vs. 71.0; *p* = 0.522). Similarly, no statistically significant differences were observed across individual domains of each questionnaire. Although some domains demonstrated minor variations—for example, higher body image scores in ulcerative colitis and higher emotional functioning scores in Crohn's disease—these differences were not statistically significant. FDR correction was also applied to the disease-type comparisons; after correction, no domain-level differences between children with Crohn's disease and ulcerative colitis remained statistically significant.

**Table 7:** Comparison of disease-specific HRQoL, generic HRQoL, and family functioning between children with Crohn's disease and ulcerative colitis in the Russian children cohort.

Variable	Crohn's disease (*n* = 59) Me [Q1; Q3]	Ulcerative colitis (*n* = 46) Me [Q1; Q3]	*p*-value
IMPACT-III			
Well-being	71.0 [60.0; 83.0]	67.0 [54.5; 80.0]	0.366
Emotional functioning	71.0 [57.0; 79.0]	68.0 [51.0; 75.0]	0.508
Social functioning	84.0 [75.0; 89.0]	80.0 [70.8; 89.0]	0.187
Body image	75.0 [63.0; 88.0]	81.0 [69.0; 92.5]	0.144
Total score	76.0 [64.0; 83.0]	70.5 [61.0; 80.8]	0.270
PedsQL			
Physical functioning	78.0 [66.0; 88.0]	75.0 [66.0; 90.3]	0.482
Emotional functioning	65.0 [50.0; 80.0]	62.5 [50.0; 80.0]	0.678
Social functioning	90.0 [77.5; 100.0]	95.0 [85.0; 100.0]	0.233
School functioning	65.0 [50.0; 80.0]	65.0 [46.3; 75.0]	0.375
Total score	73.0 [63.0; 84.0]	75.0 [64.5; 84.8]	0.681
PedsQL FIM			
Physical functioning	67.0 [52.0; 90.0]	67.0 [54.0; 83.0]	0.930
Emotional functioning	65.0 [50.0; 85.0]	55.0 [45.0; 70.0]	0.100
Social functioning	88.0 [69.0; 100.0]	81.0 [69.0; 94.0]	0.580
Cognitive functioning	80.0 [57.5; 95.0]	75.0 [66.3; 90.0]	0.988
Communication	75.0 [50.0; 92.0]	75.0 [67.0; 100.0]	0.474
Worry	55.0 [40.0; 70.0]	55.0 [40.0; 75.0]	0.809
Daily activities	75.0 [58.0; 100.0]	71.0 [50.0; 89.8]	0.777
Family relationships	95.0 [75.0; 100.0]	90.0 [75.0; 100.0]	0.901
Total score	75.0 [58.5; 83.5]	71.0 [61.0; 78.0]	0.522

IMPACT-III, disease-specific HRQoL questionnaire for children with IBD; PedsQL, Pediatric Quality of Life Inventory 4.0 Generic Core Scales; PedsQL FIM, PedsQL Family Impact Module.

### Correlation analysis

**Figure 1:**
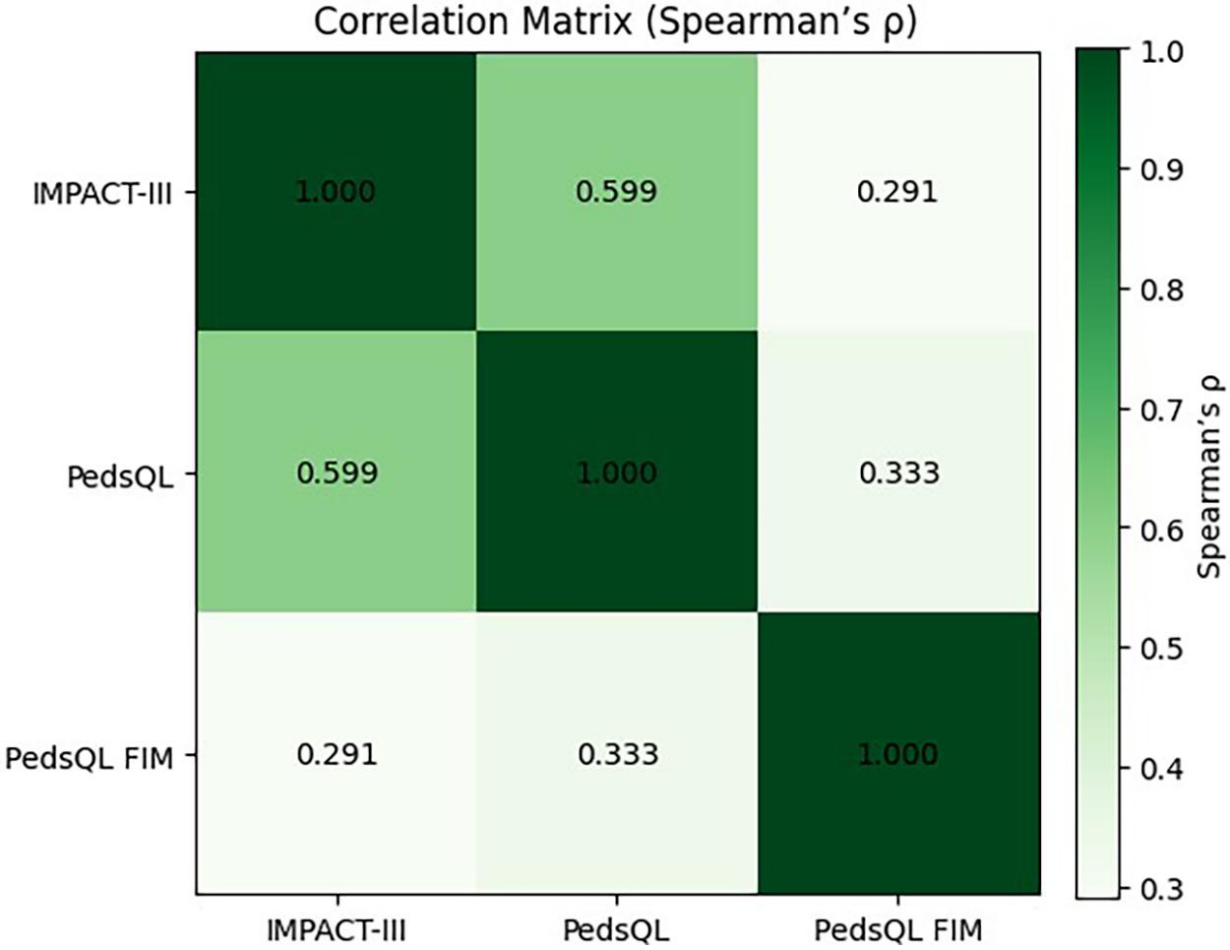
Spearman correlations among total disease-specific HRQoL, generic HRQoL, and family-functioning scores in the Russian cohort of children with IBD. IMPACT-III, disease-specific health-related quality-of-life questionnaire for children with inflammatory bowel disease; PedsQL, Pediatric Quality of Life Inventory 4.0 Generic Core Scales; PedsQL FIM, Pediatric Quality of Life Inventory Family Impact Module; IBD, inflammatory bowel disease.

Statistically significant positive correlations were observed between HRQoL measures. The total IMPACT-III score showed a moderate correlation with the PedsQL total score (*ρ* = 0.599; *p* < 0.001) and a weaker but significant correlation with family functioning as assessed by the PedsQL Family Impact Module (*ρ* = 0.291; *p* = 0.003). In addition, PedsQL scores demonstrated a moderate correlation with PedsQL Family Impact Module scores (*ρ* = 0.333; *p* < 0.001). The results of the correlation analysis are presented in Figure 1.

**Figure 2:**
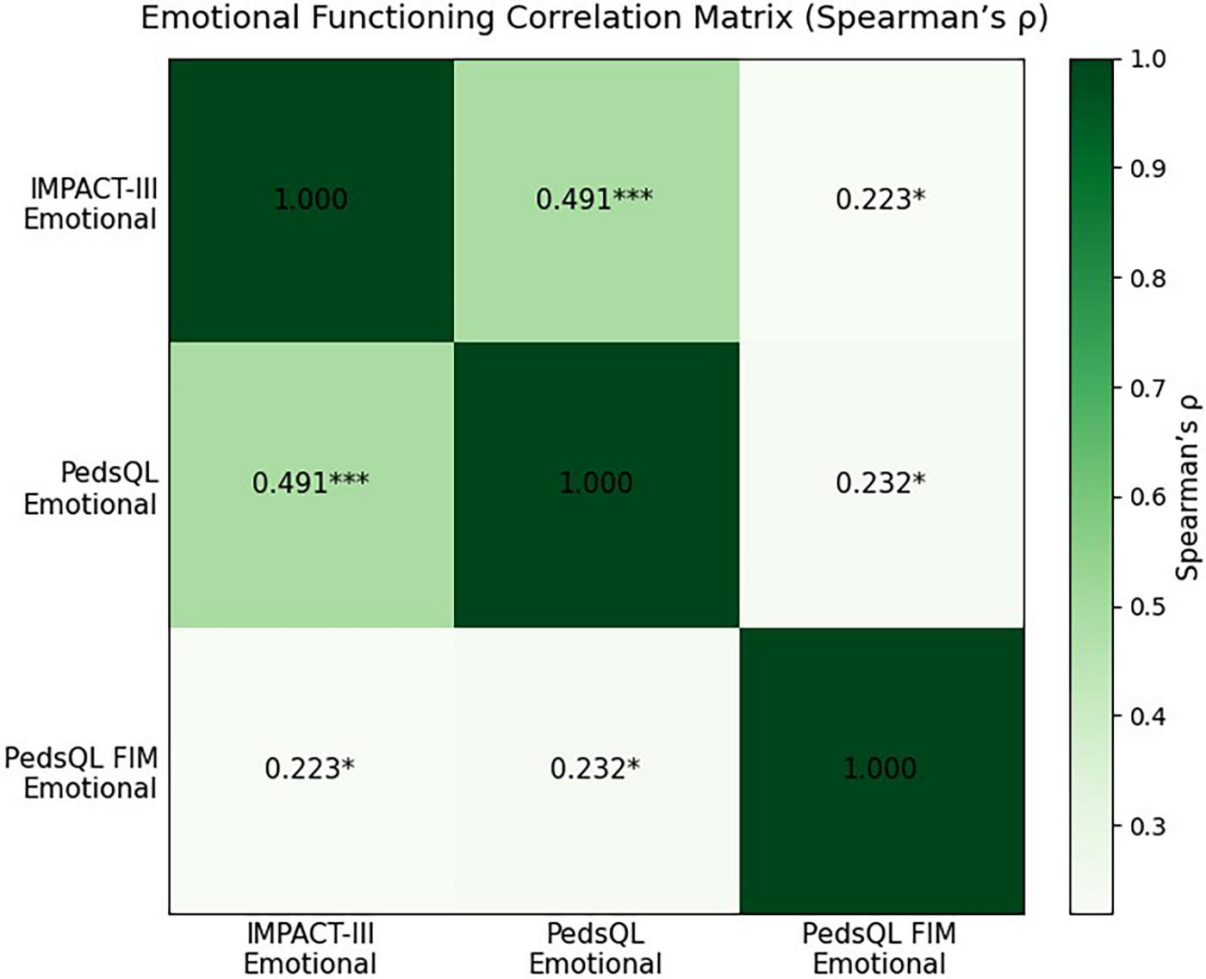
Spearman correlations among emotional-functioning domain scores from IMPACT-III, PedsQL, and PedsQL FIM in the Russian cohort of children with IBD. IMPACT-III, disease-specific health-related quality-of-life questionnaire for children with inflammatory bowel disease; PedsQL, Pediatric Quality of Life Inventory 4.0 Generic Core Scales; PedsQL FIM, Pediatric Quality of Life Inventory Family Impact Module. *p < 0.05; ***p < 0.001.

Because emotional functioning was consistently among the lowest-scoring domains, an additional domain-level correlation analysis was performed for emotional functioning subscales across the three instruments. A moderate positive correlation was observed between IMPACT-III emotional functioning and PedsQL emotional functioning (*ρ* = 0.491, *p* < 0.001). Weaker but statistically significant positive correlations were observed between IMPACT-III emotional functioning and PedsQL FIM emotional functioning (*ρ* = 0.223, *p* < 0.05), and between PedsQL emotional functioning and PedsQL FIM emotional functioning (*ρ* = 0.232, *p* < 0.05). The emotional-domain correlation matrix is presented in Figure 2.

## Discussion

In this single-center Russian cohort of children with IBD, no statistically significant differences were observed in disease-specific HRQoL, generic HRQoL, and family functioning between children with IBD receiving biologic therapy and those receiving conventional therapy. Similarly, no significant differences were observed between children with Crohn's disease and ulcerative colitis across the assessed instruments. These findings remained consistent after FDR correction for multiple domain-level comparisons. Additional sensitivity analysis restricted to children with low clinical disease activity and stratified analysis by disease type also did not identify statistically significant differences in total HRQoL scores between treatment groups. However, given the observational design, non-randomized treatment allocation, and baseline imbalances in disease type and PUCAI values, these findings should be interpreted cautiously and should not be considered evidence of equivalence between groups.

A key methodological consideration is the imbalance in disease type distribution between treatment groups. Crohn's disease was more frequent in the biologic therapy group, whereas ulcerative colitis was more frequent in the conventional therapy group. In addition, children with ulcerative colitis in the conventional therapy group had higher PUCAI scores. These imbalances may have confounded the treatment-group comparisons because disease phenotype and disease activity are important determinants of HRQoL in children with IBD. To address this limitation, we performed additional sensitivity and stratified analyses. The sensitivity analysis restricted to children with low clinical disease activity showed no statistically significant differences between treatment groups in total IMPACT-III, PedsQL, or PedsQL FIM scores. Similarly, stratified analyses performed separately in children with Crohn's disease and ulcerative colitis did not show statistically significant treatment-group differences in total HRQoL scores. Nevertheless, residual confounding cannot be excluded, particularly because subgroup sizes were limited.

Although no statistically significant between-group differences were identified, domain-specific analysis revealed consistent patterns across questionnaires. In both treatment groups, the highest scores were observed in social functioning, whereas emotional functioning was consistently one of the most vulnerable domains across both IMPACT-III and PedsQL. Children receiving biologic therapy showed a tendency toward higher emotional functioning scores, but this difference did not reach statistical significance. The additional domain-level correlation analysis further supported the relevance of emotional functioning. The strongest emotional-domain association was observed between IMPACT-III and PedsQL, suggesting convergence between disease-specific and generic child-reported emotional HRQoL. In contrast, weaker correlations with the PedsQL FIM emotional functioning domain indicate that child emotional functioning and family emotional functioning are related but distinct dimensions.

The persistence of reduced emotional functioning highlights the psychological burden of children IBD. Even when clinical disease activity is relatively controlled, children may continue to experience anxiety related to relapse, disease progression, repeated hospital visits, dietary restrictions, and long-term therapy. Adolescence is already a psychologically sensitive developmental period, and the presence of a chronic relapsing disease may further affect emotional adaptation and self-perception. Treatment-related factors, including injections, infusions, and regular monitoring, may also contribute to emotional distress; however, these factors were not directly assessed in the present study and should be evaluated in future research.

An important finding of this study is the absence of significant differences in HRQoL between Crohn's disease and ulcerative colitis. Although Crohn's disease is often associated with a more aggressive course, higher risk of complications, and greater likelihood of surgery, these clinical differences were not reflected in subjective HRQoL outcomes in the present cohort. This may be explained by modern treatment strategies, regular follow-up, and comparable psychosocial stressors shared by both groups, including chronic treatment exposure, fear of exacerbation, school disruption, and limitations in daily life.

These findings are consistent with previous studies demonstrating that HRQoL in children with IBD is more strongly influenced by disease activity and psychosocial factors than by treatment modality or disease type. *Werner* et al. (26) reported that effective disease control, rather than therapeutic strategy, is the primary determinant of HRQoL. Similarly, *Abdovic* et al. (4) showed that impairments in emotional functioning may persist even during clinical remission. Evidence from systematic reviews, including *Kunz* et al. (8), further supports the concept that HRQoL in pediatric IBD is shaped by a complex interaction of clinical and psychosocial factors. Comparable findings have also been reported in the Russian population, where no significant differences in HRQoL between disease types were observed (10).

Family functioning demonstrated a similar pattern. The highest scores in the PedsQL Family Impact Module were observed in the “family relationships” domain, whereas the lowest scores were recorded in the “worry” domain. This indicates that, despite preserved family cohesion, parents continue to experience substantial anxiety related to disease progression, treatment efficacy, long-term prognosis, and possible complications. Parental stress may influence children's illness perception, coping strategies, and adherence to therapy (8, 9). Therefore, family functioning should be considered an important component of care for children with IBD rather than a secondary psychosocial outcome.

The significant positive correlations between IMPACT-III, PedsQL, and PedsQL FIM further support the multidimensional nature of HRQoL in children with IBD. The correlation between disease-specific and generic HRQoL suggests convergence between these instruments, while the association with family functioning emphasizes the role of the family environment in shaping children's subjective well-being. The emotional-domain correlation analysis provided further insight into the emotional component of HRQoL, showing that child-reported emotional functioning and family emotional functioning are related but distinct dimensions of disease burden. These findings support the need for a multidisciplinary and family-centered approach to management of IBD in children, with particular attention to emotional well-being.

This study has several strengths, including the simultaneous assessment of disease-specific HRQoL, generic HRQoL, and family functioning using validated instruments in a real-world cohort of children with IBD. In addition, data on HRQoL in children with IBD from Eastern Europe and the Russian Federation remain limited, which increases the relevance of the present findings in the international context.

Several limitations should be acknowledged. First, the single-center, cross-sectional design does not allow assessment of causal relationships or longitudinal changes in HRQoL. Second, treatment allocation was not randomized and reflected real-world clinical practice, which may have introduced selection bias and confounding. The biologic therapy group included a higher proportion of children with Crohn's disease, whereas ulcerative colitis was more frequent in the conventional therapy group. In addition, children with ulcerative colitis in the conventional therapy group had higher PUCAI scores. Although sensitivity and stratified analyses were performed to address these imbalances, residual confounding cannot be excluded because of the observational design and limited subgroup sizes. Third, no *a priori* sample size calculation was performed. The *post-hoc* sensitivity power analysis indicated that the available sample size was sufficient to detect moderate-to-large between-group differences, but may have been underpowered to detect small differences. Therefore, the absence of statistically significant differences should not be interpreted as evidence of equivalence between treatment groups. Fourth, multiple domain-level comparisons were performed across several HRQoL and family functioning domains. Although FDR correction was applied, these domain-level analyses should still be interpreted as exploratory. Finally, endoscopic disease activity, objective inflammatory biomarkers such as C-reactive protein and fecal calprotectin, and formal psychological screening tools were not systematically included in the analysis. As a result, disease control was assessed primarily using clinical activity indices, which may not fully reflect subclinical inflammation or psychological burden. Future multicenter longitudinal studies combining HRQoL assessment with objective inflammatory markers, endoscopic data, and psychological evaluation are needed to provide a more comprehensive understanding of factors influencing quality of life in pediatric IBD.

## Conclusion

In this cross-sectional cohort of children with IBD, no statistically significant differences were observed in disease-specific HRQoL, generic HRQoL, or family functioning across treatment modalities or disease phenotypes. These findings suggest that HRQoL in children with IBD may be shaped not only by treatment strategy or disease type, but also by emotional adaptation, disease control, and family-related factors. Emotional functioning remained consistently vulnerable, while parental worry represented an important component of family burden. Routine HRQoL assessment, psychological screening, and family-oriented support should therefore be integrated into care for children with IBD to improve long-term adaptation and patient-centered outcomes.

## Data Availability

The raw data supporting the conclusions of this article will be made available by the authors, without undue reservation.

## References

[1] Wehkamp J Götz M Herrlinger K Steurer W Stange EF. Inflammatory bowel disease: crohn’s disease and ulcerative colitis. Dtsch Ärztebl Int. (2016) 113:72–82. 10.3238/arztebl.2016.0072 26900160 PMC4782273

[2] Bruner LP White AM Proksell S. Inflammatory bowel disease. Prim Care Clin Off Pract. (2023) 50:411–27. 10.1016/j.pop.2023.03.009 37516511

[3] Ahmed S Alam S Alsabri M. Health-Related quality of life in pediatric inflammatory bowel disease patients: a narrative review. Cureus. (2022) 14(9):e29282. 10.7759/cureus.29282 36277571 PMC9578282

[4] Abdovic S Mocic Pavic A Milosevic M Persic M Senecic-Cala I Kolacek S. The IMPACT-III (HR) questionnaire: a valid measure of health-related quality of life in Croatian children with inflammatory bowel disease. J Crohns Colitis. (2013) 7:908–15. 10.1016/j.crohns.2012.12.010 23333037

[5] van Rheenen PF Aloi M Assa A Bronsky J Escher JC Fagerberg UL. The medical management of paediatric crohn’s disease: an ECCO-ESPGHAN guideline update. J Crohns Colitis. (2021) 15:171–94. 10.1093/ecco-jcc/jjaa161 33026087

[6] Turner D Levine A Escher JC Griffiths AM Russell RK Dignass A. Management of pediatric ulcerative colitis: joint ECCO and ESPGHAN evidence-based consensus guidelines. J Pediatr Gastroenterol Nutr. (2012) 55:340–61. 10.1097/MPG.0b013e3182662233 22773060

[7] Chouliaras G Margoni D Dimakou K Fessatou S Panayiotou I Roma-Giannikou E. Disease impact on the quality of life of children with inflammatory bowel disease. World J Gastroenterol. (2017) 23:1067. 10.3748/wjg.v23.i6.1067 28246481 PMC5311096

[8] Kunz JH Greenley RN Howard M. Maternal, paternal, and family health-related quality of life in the context of pediatric inflammatory bowel disease. Qual Life Res. (2011) 20:1197–204. 10.1007/s11136-011-9853-3 21293931

[9] Greenley RN Hommel KA Nebel J Raboin T Li S-H Simpson P. A meta-analytic review of the psychosocial adjustment of youth with inflammatory bowel disease. J Pediatr Psychol. (2010) 35:857–69. 10.1093/jpepsy/jsp120 20123705 PMC3107587

[10] Tagirova AR Sichinava IV Savvateeva OA Borisova EV. Quality of life of children with crohn’s disease as a potential criterion for monitoring disease activity. Doctor.Ru. (2020) 19(10):27–32. 10.31550/1727-2378-2020-19-10-27-32 [in Russian].

[11] Werner H Landolt MA Buehr P Koller R Nydegger A Spalinger J. Validation of the IMPACT-III quality of life questionnaire in Swiss children with inflammatory bowel disease. J Crohns Colitis. (2014) 8:641–8. 10.1016/j.crohns.2013.11.025 24342766

[12] Uggla C Lindh V Lind T Lindkvist M. IMPACT-III is a valid and reliable questionnaire for assessing health-related quality of life in Swedish children with inflammatory bowel disease. Acta Paediatr. (2018) 107:347–53. 10.1111/apa.14119 29032599

[13] Vanhelst J Béghin L Drumez E Djeddi-Dine D Tuck D Coopman S. Validation of the IMPACT-III questionnaire in French children with inflammatory bowel disease. J Pediatr Gastroenterol Nutr. (2023) 76:e71–6. 10.1097/MPG.0000000000003716 36735394

[14] Lee GW Chew KS Wong SY Chong SY Ong SY Lee WS. Quality of life in Malaysian children with inflammatory bowel disease: an understudied population. J Paediatr Child Health. (2022) 58:1972–9. 10.1111/jpc.16130 35880617

[15] Ministry of Health of the Russian Federation. Crohn’s disease in children: clinical guidelines. (2024). Available online at: https://cr.minzdrav.gov.ru/view-cr/682_2 (Accessed October 16, 2024) [in Russian].

[16] Ministry of Health of the Russian Federation. Ulcerative colitis in children: clinical guidelines. (2024). Available online at: https://cr.minzdrav.gov.ru/view-cr/391_3 (Accessed October 16, 2024) [in Russian].

[17] Batra S Conklin LS. Therapeutics for inflammatory bowel diseases in children and adolescents: a focus on biologics and an individualized treatment paradigm. In: Kiess W Schwab M Van Den Anker J, editors. Pediatric Pharmacotherapy. Handbook of Experimental Pharmacology. Cham: Springer International Publishing (2019). p. 363–75. 10.1007/164_2019_255 PMC7371003 31342277

[18] Hoelz H Bragagna L Litwin A Koletzko S Le Thi TG Schwerd T. Pediatric IBD patients treated with infliximab and proactive drug monitoring benefit from early concomitant immunomodulatory therapy: a retrospective analysis of a 10-year real-life cohort. Inflamm Bowel Dis. (2024) 30:2004–18. 10.1093/ibd/izad277 38011813

[19] Wlazlo M Meglicka M Wiernicka A Osiecki M Matuszczyk M Kierkus J. Combination biologic therapy in pediatric inflammatory bowel disease: safety and efficacy over a minimum 12-month follow-up period. J Pediatr Gastroenterol Nutr. (2024) 79:54–61. 10.1002/jpn3.12179 38477410

[20] Illarionov AS Radygina TV Potapov AS Fisenko AP Kuptsova DG Petrichuk SV. Therapeutic drug monitoring of Adalimumab in inflammatory bowel disease in children. Vopr Det Dietol. (2021) 19:14–25. 10.20953/1727-5784-2021-3-14-25

[21] Maritime Inflammatory Bowel Disease Research Alliance. The IMPACT-III questionnaire: a valid measure of health-related quality of life in pediatric inflammatory bowel disease. Available online at: https://mirapeds.ca/miraresearch/impact/ (Accessed September 9, 2024). 10.1097/00005176-200210000-00018 12394384

[22] Otley A Smith C Nicholas D Munk M Avolio J Sherman PM. The IMPACT questionnaire: a valid measure of health-related quality of life in pediatric inflammatory bowel disease. J Pediatr Gastroenterol Nutr. (2002) 35:557–63. 10.1097/00005176-200210000-00018 12394384

[23] Grant A MacIntyre B Kappelman MD Otley AR. A new domain structure for the IMPACT-III health-related quality of life tool for pediatric inflammatory bowel disease. J Pediatr Gastroenterol Nutr. (2020) 71:494–500. 10.1097/MPG.0000000000002824 32960540

[24] Varni JW Seid M Kurtin PS. PedsQL™ 4.0: reliability and validity of the pediatric quality of life Inventory^TM^ version 4.0 generic core scales in healthy and patient populations. Med Care. (2001) 39:800–12. 10.1097/00005650-200108000-00006 11468499

[25] Varni JW Sherman SA Burwinkle TM Dickinson PE Dixon P. The PedsQL™ family impact module: preliminary reliability and validity. Health Qual Life Outcomes. (2004) 2:55. 10.1186/1477-7525-2-55 15450120 PMC521692

[26] Werner H Landolt MA Buehr P Koller R Nydegger A Spalinger J. Changes in health-related quality of life over a 1-year follow-up period in children with inflammatory bowel disease. Qual Life Res. (2017) 26:1617–26. 10.1007/s11136-017-1513-9 28197756

