# Supplementary material for: Health-Related quality of life in Russian children with inflammatory bowel disease is comparable across disease types and treatment modalities
Source: Front Pediatr. 2026 Jul 29;14:1908855. doi: 10.3389/fped.2026.1908855 (PMC13461728; doi:10.3389/fped.2026.1908855)
Supplement: Supplementary file 1 [file Table1.docx]

Supplementary Material

# 1 Supplementary Data

Benjamini–Hochberg false discovery rate correction was applied separately for:

1. **Treatment modality comparisons**: biologic vs conventional therapy (table A)
2. **Disease type comparisons**: Crohn’s disease vs ulcerative colitis (table B)

*Table A. FDR correction for treatment-modality comparisons*

| **Variable** | **Raw p-value** | **FDR-adjusted p-value** |
| --- | --- | --- |
| IMPACT-III Well-being | 0.492 | 0.999 |
| IMPACT-III Emotional functioning | 0.068 | 0.792 |
| IMPACT-III Social functioning | 0.250 | 0.792 |
| IMPACT-III Body image | 0.835 | 0.999 |
| IMPACT-III Total score | 0.136 | 0.792 |
| PedsQL Physical functioning | 0.916 | 0.999 |
| PedsQL Emotional functioning | 0.320 | 0.869 |
| PedsQL Social functioning | 0.999 | 0.999 |
| PedsQL School functioning | 0.213 | 0.792 |
| PedsQL Total score | 0.962 | 0.999 |
| PedsQL FIM Physical functioning | 0.554 | 0.999 |
| PedsQL FIM Emotional functioning | 0.527 | 0.999 |
| PedsQL FIM Social functioning | 0.586 | 0.999 |
| PedsQL FIM Cognitive functioning | 0.881 | 0.999 |
| PedsQL FIM Communication | 0.206 | 0.792 |
| PedsQL FIM Worry | 0.717 | 0.999 |
| PedsQL FIM Daily activities | 0.239 | 0.792 |
| PedsQL FIM Family relationships | 0.671 | 0.999 |
| PedsQL FIM Total score | 0.756 | 0.999 |

*Table B. FDR correction for disease-type comparisons*

| **Variable** | **Raw p-value** | **FDR-adjusted p-value** |
| --- | --- | --- |
| IMPACT-III Well-being | 0.366 | 0.902 |
| IMPACT-III Emotional functioning | 0.508 | 0.902 |
| IMPACT-III Social functioning | 0.187 | 0.902 |
| IMPACT-III Body image | 0.144 | 0.902 |
| IMPACT-III Total score | 0.270 | 0.902 |
| PedsQL Physical functioning | 0.482 | 0.902 |
| PedsQL Emotional functioning | 0.678 | 0.924 |
| PedsQL Social functioning | 0.233 | 0.902 |
| PedsQL School functioning | 0.375 | 0.902 |
| PedsQL Total score | 0.681 | 0.924 |
| PedsQL FIM Physical functioning | 0.930 | 0.982 |
| PedsQL FIM Emotional functioning | 0.100 | 0.902 |
| PedsQL FIM Social functioning | 0.580 | 0.918 |
| PedsQL FIM Cognitive functioning | 0.988 | 0.988 |
| PedsQL FIM Communication | 0.474 | 0.902 |
| PedsQL FIM Worry | 0.809 | 0.961 |
| PedsQL FIM Daily activities | 0.777 | 0.961 |
| PedsQL FIM Family relationships | 0.901 | 0.982 |
| PedsQL FIM Total score | 0.522 | 0.902 |

After FDR correction, **no disease-type comparison remained statistically significant**.
